# Acceptability and Efficacy of a Web‐Based, Intuitive Eating‐Focused Single Session Intervention for Recurrent Binge Eating: A Randomized Controlled Trial

**DOI:** 10.1002/eat.24466

**Published:** 2025-05-15

**Authors:** Mariel Messer, Claudia Liu, Matthew Fuller‐Tyszkiewicz, Cleo Anderson, Tracy L. Tylka, Jake Linardon

**Affiliations:** ^1^ SEED Lifespan Strategic Research Centre, School of Psychology, Faculty of Health Deakin University Geelong Victoria Australia; ^2^ Department of Psychology The Ohio State University Columbus Ohio USA

**Keywords:** binge eating, eating disorder, randomized controlled trial, single session intervention

## Abstract

**Objective:**

Intuitive eating is a viable intervention target for binge eating, yet current programs designed to cultivate this pattern of eating face challenges with scalability and accessibility. We developed a web‐based, intuitive eating‐focused, single‐session intervention (SSI) and evaluated its acceptability and efficacy among individuals with recurrent binge eating.

**Method:**

Two‐hundred‐forty‐eight participants reporting recurrent binge eating were randomly assigned to the SSI or a waitlist. Study assessments were conducted at baseline, 2‐week post‐test, and 6‐week follow‐up. Analyses were performed under the intention‐to‐treat principle.

**Results:**

Evidence of acceptability was observed among study retainers, with good ratings of perceived usefulness and satisfaction, and two in three stating that they would recommend the program. Issues with attrition were apparent across both groups. The intervention group reported significantly greater pre–post improvements than the control group in intuitive eating, symptoms of eating disorders, body appreciation, and body interoception, with moderate to large effect sizes. Improvements in intervention participants persisted at 6 weeks and were largely stable across sensitivity analyses that handled missing data in different ways, with a few exceptions. Between 30% and 60% of intervention participants reported that the SSI had increased indicators of confidence and motivation to change, and receptivity towards further help options.

**Conclusion:**

This study highlights the acceptability and potential efficacy of brief, but concentrated interventions designed to foster intuitive eating for binge eating. Findings add to a growing body of literature highlighting the potential benefits of SSIs for symptoms of eating disorders.

**Summary**

- Fostering intuitive eating could help reduce symptoms of eating disorders, yet current intuitive eating interventions face challenges with accessibility and scalability.
- Our web‐based, intuitive eating‐focused, single session intervention was well‐tolerated and produced significant improvements in eating disorder symptoms among individuals with recurrent binge eating.
- Single session interventions may play a valuable role in supporting people with eating disorder symptoms.

## 1 Introduction

Binge‐spectrum eating disorders—defined as binge‐eating disorder, bulimia nervosa, and their subthreshold variants—impair functioning and are associated with high rates of comorbidity and relapse (Schmidt et al. 2025). Chaotic eating habits are a defining feature of these disorders, with those affected typically cycling through periods of intentional undereating (i.e., deliberate food restriction) and loss of control overeating (i.e., objective binge eating). There is a strong body of theoretical and empirical research linking dietary restraint to the onset and persistence of binge eating (Fairburn 2008; Messer, Eckley, et al. 2024). Dietary restraint involves the conscious attempt to restrict food intake to regulate body weight and typically involves multiple demanding food rules. Dietary restraint may encourage objective binge eating through a complex interaction of physiological and psychological factors (Fairburn 2008).

Normalizing eating patterns is a key focus of established interventions designed to target binge eating (Fairburn 2008). This is typically achieved through structured regular eating, defined as eating 3 meals and 2–3 snacks every 3–4 h apart, regardless of hunger‐fullness levels. Yet, an alternative approach to normalize eating is to cultivate the principles of intuitive eating. Intuitive eating is an adaptive and flexible style of eating defined as having a strong connection with physiological cues and largely eating in response to these cues (Avalos and Tylka 2006). It involves selecting foods for enjoyment but still allowing the body to function optimally, generally relying on hunger signals to determine when and how much to eat and generally respecting satiety signals by refraining from eating when satiated (Tylka and Kroon Van Diest 2013). Since intuitive eating is inherently incompatible with dieting and is thought to disrupt the mechanisms that contribute to binge eating (i.e., all‐or‐none thinking around food, extreme hunger, etc.), it represents a promising intervention target for this population.

Although concerns have been raised that encouraging intuitive eating could be risky for those with eating disorders who have severely disrupted hunger‐fullness cues (Linardon et al. 2021), available evidence suggests otherwise. Research shows that higher intuitive eating scores consistently predict reduced odds of engaging in eating disorder behaviors over time (Hazzard et al. 2020; Linardon 2021), while other evidence demonstrates that those who made a full recovery from an eating disorder report intuitive eating levels comparable to healthy controls and higher than those who are not yet or partially recovered (Koller et al. 2020). Furthermore, a number of interventions designed to teach intuitive eating have also produced significant improvements in eating disorder risk factors and symptoms (e.g., Burnette and Mazzeo 2020), further demonstrating its utility as an appropriate intervention target.

Considerable efforts have been devoted towards developing innovative eating disorder interventions that are widely accessible, scalable, and inexpensive. One type of intervention format that meets these criteria is the online single session intervention (SSI). Typically delivered via the web, an online SSI is a brief but structured psychological program designed to be completed in one sitting (Schleider et al. 2025). It is particularly useful for those with limited time and competing commitments who may struggle to engage in or complete longer, multisession programs. SSIs can be delivered as a stand‐alone intervention option for symptom management, a tool to encourage engagement in further treatment, or a complement to traditional forms of care (Linardon and Fuller‐Tyszkiewicz 2023). Recent research shows that online SSIs targeting eating disorder symptoms are generally well‐tolerated, with participants reporting that they enjoy the experience, find the content helpful for managing symptoms, and would recommend the program to others (Smith et al. 2023). In addition, SSIs have been shown to produce significant clinical improvements, with studies reporting meaningful reductions in core symptoms such as binge eating, dietary restraint, and body dissatisfaction (Negi and Forbush 2025). Emerging evidence also suggests that SSIs may foster an increased sense of self‐efficacy to reduce binge eating, possibly because they provide participants with foundational knowledge and practical tools to support behavior change (Messer, Fuller‐Tyszkiewicz, et al. 2024).

Given the promise of intuitive eating as a viable intervention target and the potential of online SSIs to enhance treatment accessibility and uptake, we developed a web‐based, intuitive eating‐focused SSI for individuals with binge‐spectrum eating disorders. We conducted a randomized controlled trial (RCT) evaluating the acceptability and efficacy of this SSI. We assessed the SSI's impact on improving intuitive eating, symptoms of eating disorders (e.g., binge eating, dietary restriction, etc.), body appreciation, and body interoception. We included body appreciation as a secondary outcome because intuitive eating interventions promote respect for and responsiveness to the body (Burnette and Mazzeo 2020), making it reasonable to expect that improvements in body appreciation would be a natural by‐product of learning to eat intuitively. We also included body interoception as a secondary outcome because intuitive eating emphasizes tuning into internal bodily cues (e.g., hunger and fullness) making interoceptive awareness a key mechanism through which intuitive eating is developed and reinforced. We hypothesized that the web‐based SSI would be well‐tolerated and produce significant post‐test improvements in intuitive eating and eating disorder symptoms relative to a waitlist. We also hypothesize that improvements will be sustained at the 6‐week follow‐up.

## 2 Method

### 2.1 Design

We conducted a fully remote RCT comparing the effects of a web‐based intuitive eating SSI to a waitlist control group. Study assessments were conducted at baseline, 2‐week post‐test, and 6‐week follow‐up (from post‐randomization). Deakin University provided the ethical clearance, and all participants provided informed consent. The study was registered in the Australian New Zealand Clinical Trials registry (ACTRN12624000439549p).

We note three deviations to the protocol. First, we analyzed some secondary outcomes that were available for analysis but were not preregistered, including subjective binge eating, compensatory behaviors, and dietary restriction. Second, we assessed intuitive eating via the Intuitive Eating Scale Version 3 over Version 2, as the former was published after the time of pre‐registration. Third, we had an intended target sample of a minimum of 75 specified in the protocol, but a recalculated power analysis based on new evidence for effects of SSIs (see Sample Size Calculation below) indicated that a minimum of 107 per group was required.

### 2.2 Population and Recruitment

We recruited participants in October 2024 through advertisements distributed on the authors' online educational platform for eating disorders. This platform consists of a publicly accessible website (https://breakbingeeating.com/) and associated social media channels that share general information and resources about eating disorders (Linardon et al. 2020). The advertisement indicated that the study was evaluating a brief online program designed to help individuals break free from yo‐yo dieting by teaching the principles of intuitive eating. Thus, participants were aware of the program's focus on eating behaviors and the intuitive eating framework, which may have influenced the motivations of those who chose to enroll.

Eligibility criteria was deliberately kept broad to enhance ecological validity. Participants were eligible if they were aged 18 years or over, had access to the Internet, could understand the English language, and self‐reported the presence of recurrent binge eating, which was defined as engaging in at least one objective binge eating episode every 2 weeks, on average, over the past 3 months. Objective binge eating was defined as consuming a very large amount of food in a short period and at the same time feeling a sense of loss of control. This definition was communicated to participants when answering the screening item. Participants who met the full eligibility criteria commenced the baseline assessment battery.

### 2.3 Randomization

After finishing the baseline assessment, participants were randomly assigned in a 1:1 ratio using a block size of 2, based on an automated computer‐generated sequence in Qualtrics. Because the process was fully automated, future allocations were hidden from both the research team and participants.

### 2.4 Study Conditions

#### 2.4.1 Intervention

The SSI—Mindful Plate—was a self‐guided program delivered through a secure, password‐encrypted web platform. Content was grounded in the evidence‐based framework of intuitive eating by Tribole and Resch (2020) which draws on cognitive, behavioral, and mindfulness approaches, and was delivered in multimedia formats, including written text, short videos, and visual infographics (see Figure S1). The program was designed to be completed in one sitting and in under an hour, but it was possible for users to go back and revisit content and take as long as they liked. While the program itself could not be downloaded, participants retained ongoing access to the content via the secure portal by logging in again, and were able to download the accompanying homework worksheets for offline use.

The program consisted of five psychoeducational “mini modules”: (1) getting to know your hunger, (2) fostering a healthy relationship with food, (3) feeling full and satisfied, (4) emotion regulation and body appreciation, and (5) getting moving and honoring your health. Participants were encouraged to work through each module at their own pace by watching a short video (2–5 min) and completing the corresponding activity in the follow‐along worksheets provided (Figure S2). Activities were brief and designed to teach users practical skills and thought patterns that can support and foster intuitive eating habits. For example, one activity used a CBT technique to help users develop a non‐judgmental view of food by learning how to reframe negative thoughts and beliefs. In this task, participants were asked to write down a negative thought, question its truth and accuracy, and then reframe it with a more neutral tone. Participants were encouraged to apply the strategies taught in the program to their daily life. Each module took approximately 10 min to complete. Optional supplementary “take‐home” materials were made available at the end of the program for those interested in further enhancing their knowledge on intuitive eating.

The content for the intervention was guided by the evidence‐based framework outlined in The Intuitive Eating Workbook (Tribole and Resch 2020). Given the constraints of delivering a brief, single‐session format, the author team engaged in a systematic content selection process to identify the most essential principles to emphasize. Priority was given to core components such as rejecting the diet mentality, honoring hunger, and feeling fullness, which we considered foundational to the intuitive eating approach. Other principles, including discovering the satisfaction factor and gentle nutrition, were covered more briefly due to space limitations. The content was adapted using original language, examples, and exercises, and the author team's expertise in intuitive eating research helped ensure fidelity to the core tenets of the framework.

While both mindfulness‐based interventions and intuitive eating emphasize awareness of internal cues and emotion regulation, intuitive eating places greater emphasis on rejecting dieting and restrictive eating mentalities through brief cognitive exercises. It also adopts a broader scope by incorporating strategies that foster body appreciation and encourage enjoyable physical activity, which are not core components of traditional mindfulness interventions (Kristeller et al. 2014).

#### 2.4.2 Control

The control group comprised a waitlist. Participants allocated to the control group were given access to the program after completing the 2‐week post‐test assessment.

### 2.5 Assessments

The assessment battery took approximately 15 min to complete. Participants were not compensated for completing any of the assessments.

#### 2.5.1 Baseline Characteristics

Participants were asked to report their age, gender, and ethnicity. They were also asked to indicate whether they had a current or prior diagnosis of an eating disorder, whether they were currently receiving professional help for eating or body image concerns, and whether they had prior experience in using a digital mental health intervention.

#### 2.5.2 Primary Outcomes

The primary outcomes were intuitive eating, eating disorder psychopathology, and objective binge eating. Intuitive eating was assessed using the 12‐item Intuitive Eating Scale‐3 (Tylka et al. 2024). Participants indicate their level of agreement on items assessed on a 5‐point scale, with higher scores reflecting higher intuitive eating (αs were > 0.80 in this sample). The IES‐3 has demonstrated good psychometric properties, including convergent and incremental validity, test retest reliability, and internal consistency (Tylka et al. 2024). Eating disorder psychopathology was assessed using the total score from the attitudinal items of the ED‐15, which is the average score of the shape/weight concerns subscale (e.g., “over the past week, how often have I avoided activities or people because of the way I look”) and the eating concerns subscale (“over the past week, how often have I been preoccupied with thoughts of food and eating”) derived from factor analytic techniques (Tatham et al. 2015). Items are rated on a 7‐point scale, are assessed in relation to the past week, and are averaged to produce a total score, with higher scores reflecting higher psychopathology (αs > 0.85). The ED‐15 has demonstrated internal consistency and convergent validity via its strong correlation with the full EDE‐Q (Tatham et al. 2015). Objective binge eating was assessed through a single item derived from the ED‐15, where participants were asked to report the number of episodes experienced (consuming a large amount of food in a short period, accompanied by a sense of loss of control) over the past week.

#### 2.5.3 Secondary Outcomes

Secondary outcomes included the frequency of subjective binge eating, dietary restriction, and compensatory behaviors (sum of self‐induced vomiting, driven exercise, and laxative use) experienced over the past week. Each was assessed through a single item derived from the ED‐15. Body appreciation was also a secondary outcome assessed by the 3‐item version of the Body Appreciation Scale‐2 Short‐Form, which has a near perfect correlation with its full form, has a well‐replicated unidimensional structure, and has demonstrated evidence of convergent and incremental validity (Tylka et al. 2022). Items are rated along a 5‐point scale and averaged to produce a total score, with higher scores reflecting higher respect for one's body (αs > 0.90 in this sample). Body interoception (ability to perceive and interpret bodily sensations correctly) was assessed using the 3‐item body listening subscale from the Multidimensional Assessment of Interoceptive Awareness‐2 (Mehling et al. 2018). Items are rated along a 6‐point scale and are averaged to produce a total score, with higher scores reflecting body interoception (αs > 0.88 in this sample). The body interoception subscale has demonstrated internal consistency, convergent validity, and incremental validity (Mehling et al. 2018).

#### 2.5.4 Readiness to Change

Intervention participants also answered a series of self‐created items at post‐test designed to assess level of agreement towards the program's ability to enhance readiness for change. Items asked participants whether the program (1) “increased my confidence to reduce disordered eating patterns”; (2) “increased motivation to change my disordered eating patterns”; (3) “motivated me to seek further help to address any disordered eating patterns”; and (4) “increased my receptivity towards exploring further digital mental health interventions”. Response options included *Strongly Disagree*, *Disagree*, *Neither Disagree nor Agree*, *Agree*, and *Strongly Agree*.

#### 2.5.5 Acceptability Measures

Participants reported how much of the program they accessed (response options were 0%, 25%, 50%, 75%, or 100%) and how long they spent on the program (response options were 0 min, 1–30 min, 31–60 min, or > 60 min). They were also asked to indicate whether they would recommend the program to others (*Yes*, *No*, or *Undecided*) and how useful and satisfied they were with the program (10‐point scale, ranging from *not at all* to *extremely*). Participants also indicated their level of agreement (*Strongly Disagree, Disagree, Neither Agree nor Disagree, Agree, or Strongly Agree*) regarding how engaging the text, graphics, and videos were on a 5‐point scale.

### 2.6 Sample Size

The required sample size was powered with the following assumptions: (1) a medium post‐test between‐group difference (*d* = 0.50); (2) power set to 0.80; (3) alpha set to 0.05 (two‐tailed); (4) attrition rate of 40%; and (5) allocation ratio of 1:1. Under these assumptions, a sample of 107 per group was required.

### 2.7 Data Analyses

Analyses were performed using Stata version 18. In line with intention‐to‐treat principles, participants were analyzed based on their original group assignment at baseline. Group differences at baseline for outcome variables and other available baseline measures are presented, with *t*‐tests to compare intervention and control groups on these variables. These analyses are conducted to characterize our groups and are not intended for post hoc selection of covariate adjustment, which can lead to biased estimation of treatment effects (Holmberg and Andersen 2022).

Mixed models were employed to assess the effectiveness of the intervention on primary and secondary outcomes, with time coded for comparisons with baseline as reference for immediate post‐test assessments (baseline = 0, post‐intervention = 1), group coded with waitlist as reference (waitlist control = 0, intervention = 1), and the group × time interaction terms as tests of efficacy of intervention relative to the waitlist control group at post‐test. For tests of stability of change (2–6 weeks for intervention group) models were run for the intervention group. A Gaussian distribution was assumed for all outcomes, except for the eating disorder behavioral outcomes, which were analyzed using a Poisson distribution.

In these models, missing data were addressed using multiple imputation with 50 imputations, with all baseline variables included in imputation under an inclusive auxiliary variable strategy (Mainzer et al. 2024). However, since multiple imputation assumes that missingness is ignorable (i.e., missing at random or missing completely at random)—an assumption that cannot be directly tested—sensitivity analyses were performed to assess whether the results remained robust under potential non‐ignorable missing data patterns (not missing at random; NMAR). Sensitivity analyses for the primary outcomes were conducted using pattern mixture models via the mimix package (Cro et al. 2016). Several plausible NMAR scenarios were tested, including: (1) last mean carried forward (LMCF; assumes missing values equal the group mean at last observation), (2) jump to reference (J2R; assumes missing values follow the control group's trajectory), and (3) copy increments in reference (CIR; assumes change from dropout mirrors control group's change). Each model included 50 imputations. Standardized mean differences were calculated per Feingold (2017) for continuous outcomes, and incidence rate ratios (IRRs) were used for count outcomes.

## 3 Results

### 3.1 Participant Characteristics

**FIGURE 1:**
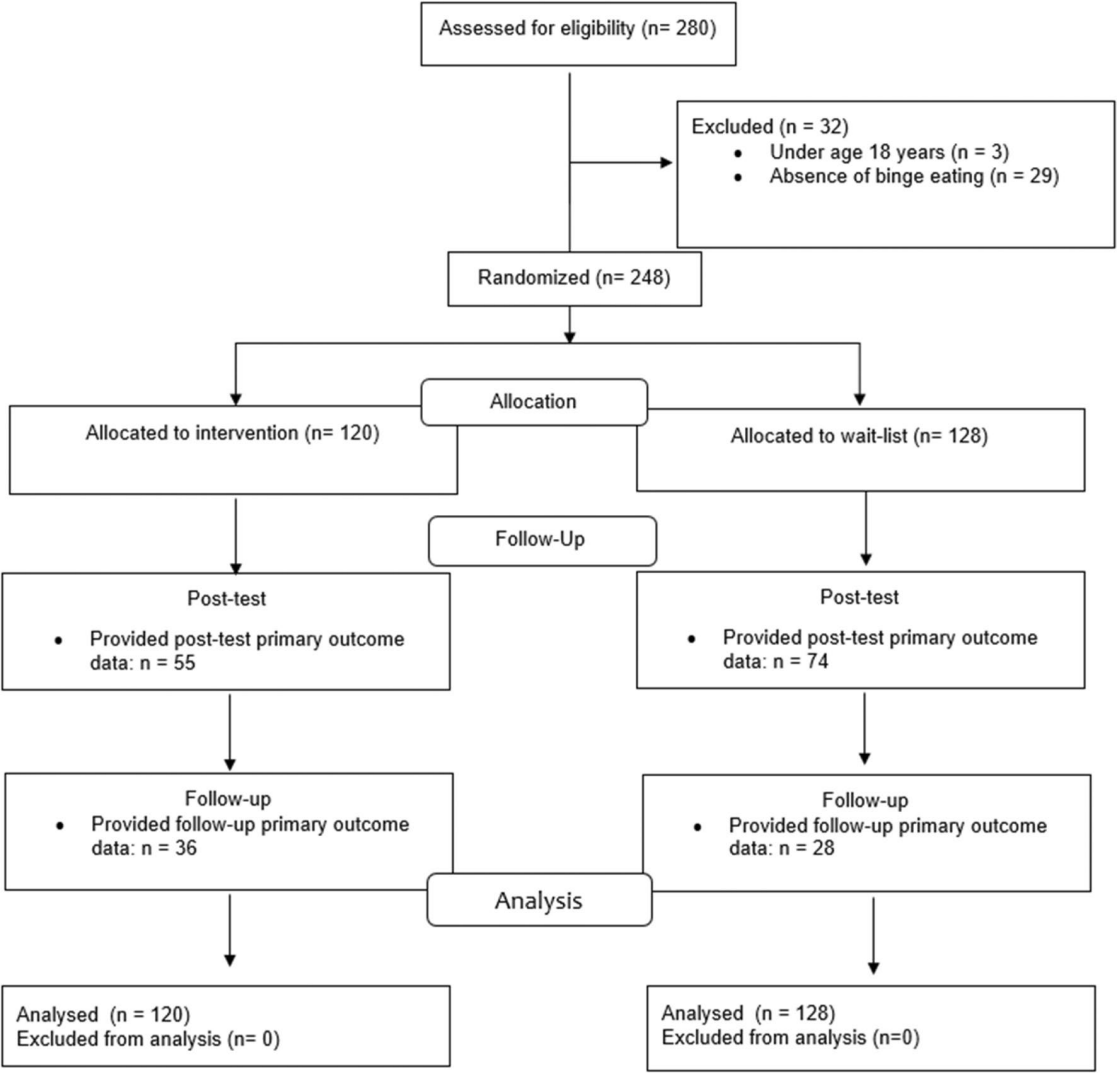
Flow of Participants throughout the study.

The randomization process is presented in Figure 1. There were 248 participants who met inclusion criteria and were randomized. The gender distribution was 91.1% (*n* = 226) women, 8.5% men (*n* = 21), and 0.4% (*n* = 1) gender non‐conforming. The ethnic distribution was 82.7% White (*n* = 205), 4.4% Asian (*n* = 11), 3.2% Hispanic (*n* = 8), 1.2% African American (*n* = 3), 0.4% Aboriginal/Torres Strait Islander (*n* = 1), 3.6% mixed (*n* = 9), and 4.4% other (*n* = 11). Among the total sample, the mean age was 41.18 years (SD = 11.60), one‐third self‐reported (*n* = 88; 35.5%) current help‐seeking or a prior (*n* = 91; 36.7%) or current (*n* = 74; 29.8%) ED diagnosis, and 49% (*n* = 122) reported experience with digital mental health interventions. The two conditions were compared on baseline variables (Table 1). Intervention participants reported higher eating disorder psychopathology, subjective binge eating episodes, and compensatory behavior episodes than waitlist participants; although effect sizes were small.

**TABLE 1:** Group comparisons on baseline variables.

Variable	Waitlist (*n* = 128)	Intervention (*n* = 120)	Test statistic	*p*	ES^a^
Age	42.57 (11.48)	39.72 (11.61)	1.95	0.053	0.25
Gender (women)	115 (90%)	111 (93%)	1.24	0.537	0.07
Ethnicity (White)	109 (85%)	96 (80%)	7.76	0.257	0.18
Prior self‐reported ED diagnosis	48 (38%)	43 (36%)	0.07	0.786	0.02
Current self‐reported ED diagnosis	41 (32%)	33 (28%)	0.61	0.436	0.49
Current help‐seeking	45 (35%)	43 (36%)	0.01	0.911	0.01
Digital mental health experience	67 (52%)	55 (46%)	1.05	0.305	0.31
Intuitive eating (IES‐3 total)	2.20 (0.54)	2.11 (0.58)	1.26	0.208	0.16
ED psychopathology (ED‐15)	4.17 (1.06)	4.43 (0.86)	2.17	0.031	0.28
Objective binge eating	7.14 (5.42)	8.59 (8.49)	1.61	0.108	0.21
Subjective binge eating	5.22 (6.46)	7.35 (8.60)	2.22	0.027	0.28
Dietary restriction	5.53 (8.82)	8.14 (12.79)	1.88	0.061	0.24
Compensatory behaviors	3.11 (6.32)	5.98 (13.71)	2.14	0.034	0.27
Body appreciation (BAS‐SF‐3)	2.26 (0.70)	2.14 (0.77)	1.33	0.186	0.17
Body interoception (MAIA‐2)	2.76 (1.02)	2.75 (1.08)	0.10	0.921	0.01

^a^ Categorical outcomes tested with chi square and ES is phi; continuous outcomes tested with *t*‐tests and ES is Cohen's *d*.

### 3.2 Study Attrition

There were 129 (52.0%) participants who provided data on primary outcomes at post‐test while 64 (25.8%) provided these data at follow‐up. Fifty‐nine (23.8%) participants completed both the post‐test and follow‐up assessment, 70 (28.2%) completed only the post‐test assessment, and 5 (2.0%) only completed the follow‐up assessment. Attrition rates were not significantly different between the groups at post‐test (*χ* ^2^ = 3.56, *p* = 0.059, Cramér's V = −0.12). For the total sample, those who dropped out at post‐test had higher body appreciation (*p* = 0.010, *d* = 0.33) and body interoception (*p* = 0.007, *d =* 0.35) scores than those who did not drop out, while those who dropped out at follow‐up were older (*p* = 0.008, *d* = 0.39). Within the control group, no baseline differences emerged between those who did versus did not drop out at post‐test, while dropouts at follow‐up reported a higher frequency of compensatory behaviors at baseline (*p* < 0.001, *d* = 0.38). Within the intervention group, none of the variables at baseline differed between dropouts and completers at post‐test and follow‐up (*p*s > 0.05). None of the other baseline variables (demographics, outcome measures) were associated with attrition overall and by group.

### 3.3 Efficacy Tests

The mean differences in intuitive eating (*d* = 0.65; 95% CI = 0.46, 0.84), eating disorder psychopathology scores (*d* = −1.07; 95% CI = −1.38, −0.76), and objective binge eating (RR = 0.61; 95% CI = 0.41, 0.89) between the two conditions in the ITT analyses were statistically significant. Significant mean differences at post‐test were also found for each secondary outcome, except for subjective binge eating. In all instances, the intervention group experienced greater improvements than the control group (Table 2).

**TABLE 2:** Means, standard deviations, and change scores on primary and secondary outcomes for study conditions.

	Study condition				Difference in change score			Change from post to follow‐up (intervention group)		
	Control		Intervention		Intervention—control					
Outcome	*n*	*M* (SD)	*n*	*M* (SD)	*M* (95% CI)	ES (95% CI)	*p*	M change	*p*	ES (95% CI)
Intuitive eating (IES‐3 total)										
Baseline	128	2.20 (0.54)	120	2.11 (0.58)						
Post‐intervention	74	2.26 (0.53)	55	2.83 (0.78)	0.65 (0.46, 0.84)	1.16 (0.82, 1.50)	< 0.001			
Follow‐up	28	3.67 (2.63)	36	2.89 (0.88)				−0.03 (−0.24, 0.17)	0.752	−0.04 (−0.31, 0.22)
ED psychopathology (ED15)										
Baseline	128	4.17 (1.06)	120	4.43 (0.86)						
Post‐intervention	74	3.89 (1.16)	55	3.00 (1.19)	−1.07 (−1.38, −0.76)	−1.10 (−1.42, −0.78)	< 0.001			
Follow‐up	27	3.07 (1.27)	36	2.86 (1.26)				−0.03 (−0.27, 0.22)	0.836	−0.03 (−0.23, 0.18)
Objective binge eating										
Baseline	128	7.14 (5.42)	120	8.59 (8.49)						
Post‐intervention	74	5.49 (5.40)	55	3.91 (5.25)	−0.49 (−0.86, −0.12)	0.61 (0.41, 0.89)	0.010			
Follow‐up	27	3.92 (5.94)	36	3.58 (5.13)				−0.31 (−0.85, 0.23)	0.261	0.73 (0.43, 1.26)
Subjective binge eating										
Baseline	128	5.21 (6.46)	120	7.35 (8.60)						
Post‐intervention	74	3.74 (5.14)	55	3.58 (4.97)	−0.45 (−0.92, 0.02)	0.64 (0.40, 1.01)	0.056			
Follow‐up	27	3.03 (4.31)	36	3.03 (3.78)				−0.12 (−0.69, 0.45)	0.684	0.89 (0.50, 1.57)
Dietary restriction										
Baseline	128	5.53 (8.82)	120	8.14 (12.79)						
Post‐intervention	74	5.05 (6.61)	55	3.02 (5.79)	−0.91 (−1.48, −0.33)	0.40 (0.23, 0.72)	0.002			
Follow‐up	27	2.59 (2.99)	36	3.11 (5.69)				0.26 (−0.57, 1.10)	0.537	1.30 (0.56, 3.00)
Compensatory behaviors										
Baseline	128	3.11 (6.32)	120	5.98 (13.71)						
Post‐intervention	74	3.04 (8.64)	55	1.29 (3.47)	−1.32 (−2.05, −0.59)	0.27 (0.13, 0.55)	< 0.001			
Follow‐up	27	0.92 (1.92)	36	1.94 (3.99)				1.04 (−0.57, 2.65)	0.207	2.82 (0.56, 14.10)
Body appreciation (BAS‐3SF)										
Baseline	128	2.26 (0.70)	120	2.14 (0.77)						
Post‐intervention	73	2.36 (0.77)	55	2.72 (0.88)	0.44 (0.24, 0.64)	0.60 (0.33, 0.87)	< 0.001			
Follow‐up	27	2.59 (0.96)	36	2.74 (0.87)				0.06 (−0.12, 0.24)	0.524	0.07 (−0.14, 0.27)
Body interoception (MAIA‐2)										
Baseline	128	2.76 (1.02)	120	2.75 (1.08)						
Post‐intervention	73	2.80 (0.98)	54	3.49 (1.06)	0.66 (0.34, 0.98)	0.63 (0.32, 0.93)	< 0.001			
Follow‐up	27	3.13 (1.23)	36	3.66 (0.85)				0.11 (−0.12, 0.33)	0.361	0.10 (−0.11, 0.31)

*Note: M* and SD values are based on non‐imputed data; mean differences and effect sizes are derived from ITT analysis using multiple imputation. effect size, *d*, for all outcomes except OBE, SBE, compensatory behaviors, and restriction (which presents rate ratio).

Table 2 presents the results from the ITT analyses on the degree of change from post‐test to follow‐up among intervention group participants. Non‐significant, negligible changes were found across all primary and secondary outcomes, indicating the stability of improvements observed at the post‐test assessment.

### 3.4 Sensitivity Analyses

Table S1 presents the sensitivity analyses on primary and secondary outcomes using different methods of handling missing data. Results remained largely consistent across models but with some minor exceptions: effects on count outcomes became non‐significant with the LMFC and CIR approach, although all other outcomes remained significant.

### 3.5 Readiness for Change

Fifty intervention participants responded to items at post‐test assessing their level of agreement towards the impact of the intervention on readiness to change indicators. Of these, 44% (*n* = 22) either “agreed” or “strongly agreed” that the intervention increased their confidence in their ability to change, 64% (*n* = 32) “agreed” or “strongly agreed” that the intervention increased their motivation for change, 32% (*n* = 16) “agreed” or “strongly agreed” that the intervention motivated them to seek further help, and 60% (*n* = 30) “agreed” or “strongly agreed” that the intervention increased their receptivity towards exploring further digital mental health interventions.

### 3.6 Usage and Acceptability

Nearly half of the intervention participants reported accessing 100% of the program, while a further 1 in 5 reported accessing more than 50% of the program. One in five reported that they spent longer than 60 min on the program, while one in four indicated that they spent between 30 and 60 min on the program. Of the intervention participants who completed post‐test acceptability measures (*n* = 53), 68% reported that they would recommend the program. The mean program usefulness rating (1–10 range) was 6.26 (SD = 2.47), and the mean program satisfaction rating (1–10 range) was 6.67 (SD = 2.36). 62% (*n* = 49), 60% (*n* = 47), and 59% (*n* = 46) either “agreed” or “strongly agreed” that the text, graphics, and video recordings were engaging, respectively.

## 4 Discussion

We evaluated the acceptability and preliminary efficacy of a newly developed web‐based intuitive eating‐focused SSI for individuals with recurrent binge eating. Evidence of acceptability was found among completers, with relatively high perceived usefulness and satisfaction ratings. But only two in three stated that they would recommend the program, which is lower than the 80% acceptability rate that has been reported previously in eating disorder‐focused SSI research (Smith et al. 2023). Study dropout was considerable and was similar to what was observed in other SSI trials conducted on the same target population (Messer, Fuller‐Tyszkiewicz, et al. 2024) but higher than what has been reported in other SSI trials of populations with different mental health problems (e.g., ~70%; Schleider et al. 2022). The SSI also led to significant moderate‐to‐large post‐test improvements in intuitive eating, various symptoms of eating disorders, and positive body image relative to the waitlist. Critically, the degree of improvement in outcomes was sustained at 6 weeks. However, for some outcomes such as binge eating and compensatory behaviors, group differences became non‐significant under certain sensitivity analyses. This was not unexpected, as these conservative approaches assume no improvement among participants lost to follow‐up, thereby providing a lower‐bound estimate of treatment effects. Furthermore, a considerable proportion of completers agreed that the program enhanced numerous indicators of readiness to change, highlighting the versatility of SSIs as not only tools for symptom management but also as catalysts for fostering engagement and preparedness for further treatment.

The present study adds to a growing body of literature demonstrating the utility of intuitive eating as a viable intervention target for binge‐spectrum eating disorders (Koller et al. 2020). We show that it is feasible to cultivate intuitive eating principles in this population by administering a brief but concentrated intervention program that is grounded in an evidence‐based framework. Our intuitive eating SSI led to significant reductions in eating disorder symptoms in both the short and longer term. There are several potential explanations for these observed benefits. One could be that the intervention helped shift participants away from a restrictive dieting mindset, reducing the physiological and psychological deprivation that triggers uncontrollable eating and the psychological rigidity that encourages other disordered eating behaviors (Fairburn 2008). An alternative explanation could be that the intuitive eating intervention takes a holistic approach to health by promoting body respect and enjoyable physical movement, which may help reduce preoccupation with body image, lessen an overvaluation of weight and shape, and mitigate compulsive exercise patterns (Tylka and Wood‐Barcalow 2015).

This study adds to an emerging evidence base on SSIs for eating disorders, demonstrating their potential to increase help‐seeking and service uptake. Our results highlight the versatility of this mode of intervention delivery, potentially serving as a suitable initial step for self‐management of symptoms. Their brevity, flexibility, and scalability suggest that SSIs may be appropriate for those who cannot receive immediate access to other help options or who face significant time constraints or competing commitments (Schleider et al. 2025). SSIs may also serve as valuable tools for fostering buy‐in to additional treatment, as they can help establish foundational knowledge necessary for change while increasing motivation, confidence, and openness to seeking further support in some users. Alternatively, SSIs may be used as “booster sessions” during or after standard treatment, reinforcing key therapeutic concepts and helping individuals maintain progress by providing accessible resources when challenges arise.

It is important to acknowledge the limitations of this study. First, we encountered problems with study retention, which was anticipated given the fully remote nature of this trial. Although attrition rates were consistent with those observed in other digital intervention trials (Messer, Fuller‐Tyszkiewicz, et al. 2024), we found evidence of potential attrition bias, in that participants who dropped out had higher body appreciation and interoceptive awareness at baseline and were older. These findings suggest that individuals who feel more positively about their bodies or more attuned to bodily cues may be less motivated to continue engaging with an intervention focused on improving those domains, potentially because they already exhibit these skills and do not feel the need to acquire content directed at further cultivating them. Similarly, older participants may face unique barriers to sustained engagement, such as lower digital literacy, reduced comfort with web‐based platforms, or competing life responsibilities that limit time available for intervention use. These patterns are in line with prior research showing that perceived relevance of content and digital literacy can influence dropout in online interventions (McClure et al. 2024). Future studies may benefit from trialing possible strategies to enhance participant retention, such as sending personalized reminders, embedding motivational prompts throughout the program, and offering brief personalized feedback on completed homework tasks to enhance user engagement and accountability.

Second, we relied on participant self‐report for collecting outcome data. While self‐report scales are mainstream in digital health research given that they facilitate rapid recruitment at minimal costs, it is important to be aware of the limitations of this method, namely the potential for over‐reporting symptom improvement and the possibility of misclassification when establishing inclusion criteria based solely on participant‐reported symptoms (Cuijpers et al. 2010). Further, we did not assess participants' engagement with other interventions during the trial, limiting our ability to determine whether external treatment contributed to outcomes. While randomization likely balanced this factor across groups, future studies should consider measuring concurrent treatment seeking to better account for its potential impact.

Third, data on readiness for change, usage, and acceptability were only collected from participants who completed assessments. Thus, we were unable to capture the perspectives of those who disengaged, which may have provided important insights into barriers to engagement or reasons for dropout.

Fourth, assessment time points were scheduled relative to randomization rather than actual program access, as the intervention platform did not record log‐in times. This means that some participants may have completed assessments without fully engaging with the program, potentially underestimating its true effects. Future studies should incorporate backend tracking to better align assessment timing with intervention use and more accurately capture outcome changes.

Fifth, the sample was predominantly female, which limits the generalizability of findings to other genders. Although binge eating affects individuals across the gender spectrum (Mitchison et al. 2017), our recruitment platform primarily reaches women, which may explain the skewed distribution. Future studies should consider targeted recruitment strategies to engage more diverse participants.

Building on the promising findings of this study, several avenues for future research may enhance the design and delivery of SSIs for binge eating. Future research should explore ways to enhance engagement and accountability in SSIs, as lack of interaction or follow‐up may contribute to high attrition. Incorporating light‐touch features such as optional therapist‐led question and answer sessions or tailored, automated feedback may help sustain user involvement without compromising scalability. In addition, combining intuitive eating with other complementary targets (e.g., self‐compassion) may enhance the intervention's impact, particularly by helping users respond constructively to perceived lapses in eating behavior. These directions may inform the development of more personalized and effective SSI models that retain their brevity while increasing reach and impact.

The present study is the first to demonstrate the acceptability and preliminary efficacy of a web‐based, intuitive eating‐focused SSI for individuals with recurrent binge eating. We show that the SSI was well‐tolerated among trial completers and led to significant improvements in intuitive eating, various symptoms of eating disorders, and positive body image. Given these promising findings, we encourage future research to more rigorously examine the utility and long‐term efficacy of SSIs for eating disorders, as their scalable and flexible nature may make them a valuable form of support that can help bridge gaps in care, enhance treatment engagement, and provide ongoing symptom management.

## Author Contributions

**Mariel Messer:** conceptualization, funding acquisition, writing – original draft, methodology, writing – review and editing. **Claudia Liu:** conceptualization, methodology, project administration, investigation, writing – review and editing. **Matthew Fuller‐Tyszkiewicz:** conceptualization, formal analysis, supervision, writing – review and editing. **Cleo Anderson:** conceptualization, investigation, writing – review and editing. **Tracy L. Tylka:** conceptualization, writing – review and editing. **Jake Linardon:** conceptualization, writing – original draft, writing – review and editing, methodology, formal analysis, supervision.

## Conflicts of Interest

The authors declare no conflicts of interest.

## Supporting information


**Data S1.** Supporting Information.

## Data Availability

The data that support the findings of this study are available from the corresponding author upon reasonable request.
