# Supplementary material for: Acceptability and Efficacy of a Web‐Based, Intuitive Eating‐Focused Single Session Intervention for Recurrent Binge Eating: A Randomized Controlled Trial
Source: Int J Eat Disord. 2025 May 15;58(8):1547–57. doi: 10.1002/eat.24466 (PMC12336771; doi:10.1002/eat.24466)
Supplement: Supplementary file 1 — Data S1. Supporting Information. [file EAT-58-1547-s001.docx]

**Figure 1**

*Screenshot of the Mindful Plate Intervention*

**
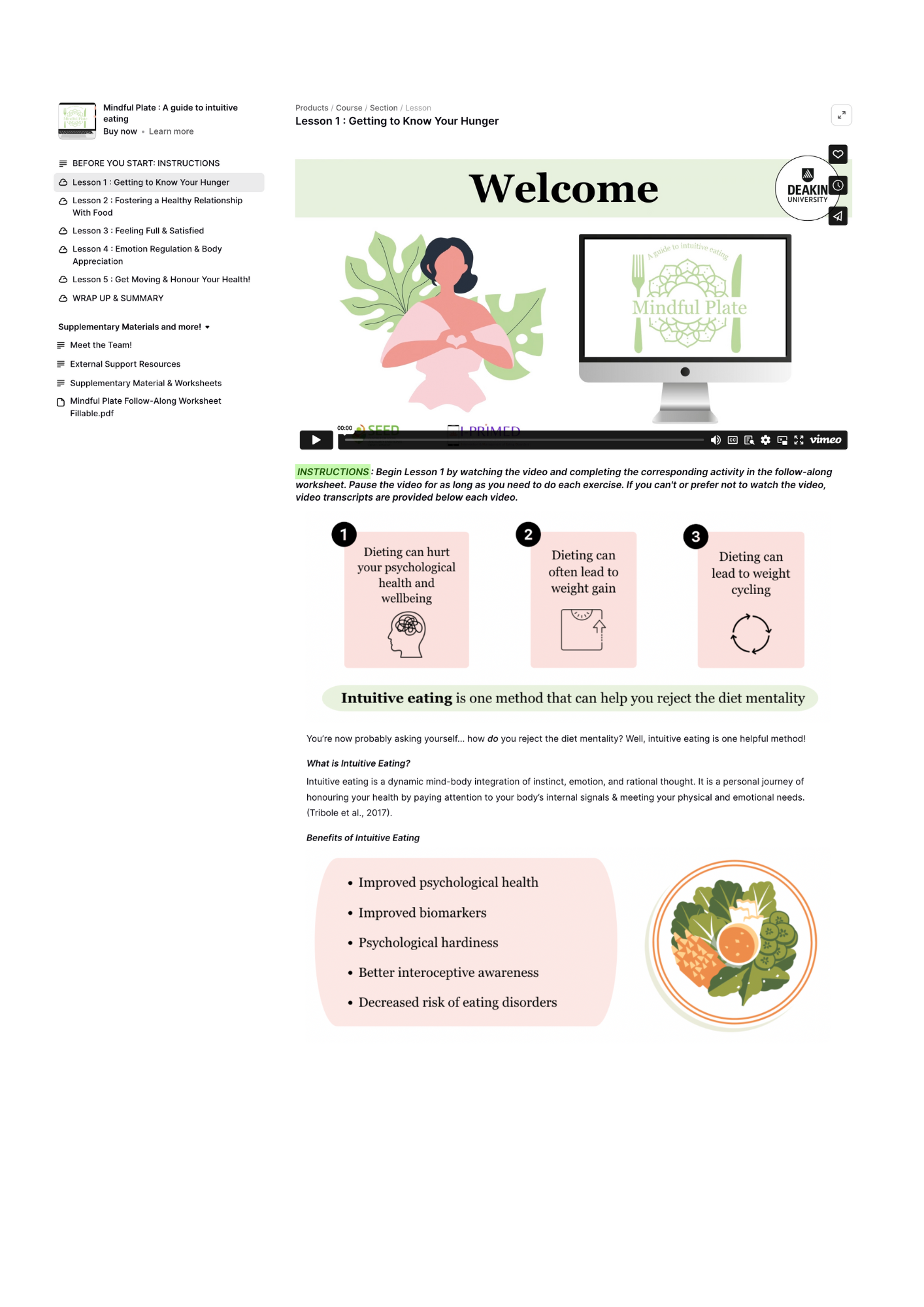
Figure 2**

*Screenshot of follow-along* **
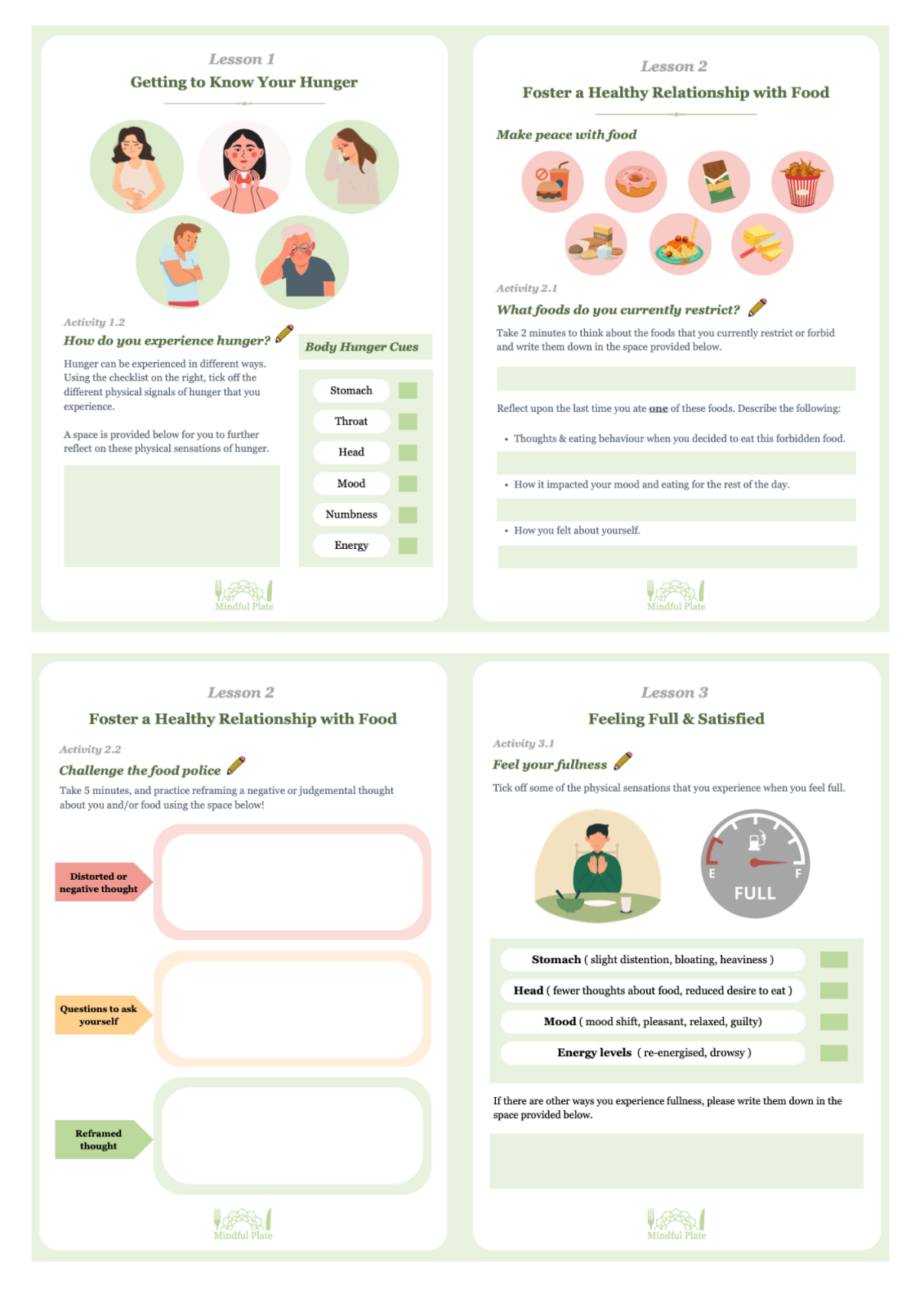
***worksheets*

**Figure 3**

*Screenshot of optional supplementary ‘take-home’ materials*

**
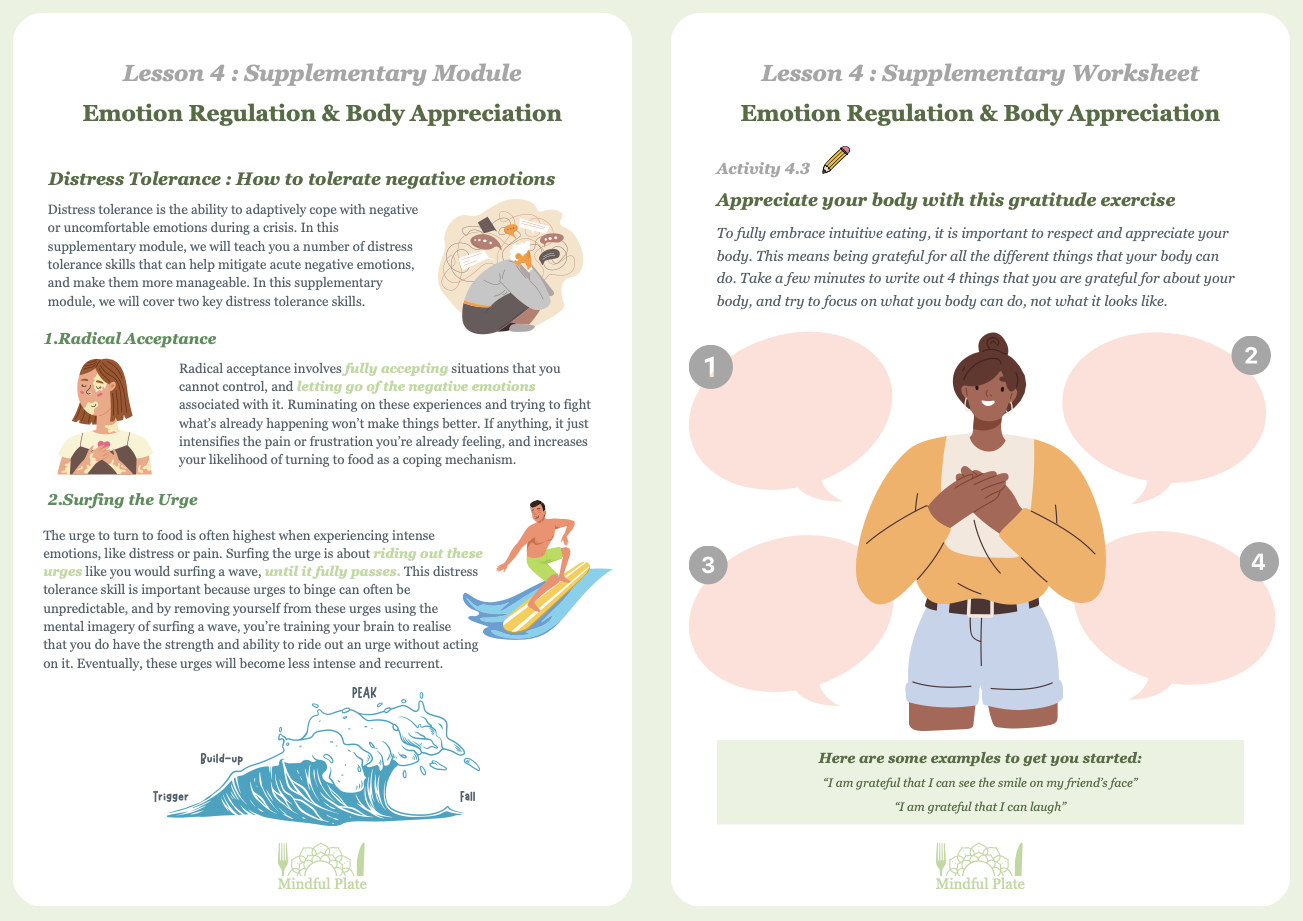
**

| Table S1  Sensitivity Analyses for Post-Intervention Effects of Intervention Vs. Control | | | | | | | | | | | |
| --- | --- | --- | --- | --- | --- | --- | --- | --- | --- | --- | --- |
|  | **LMFC approach** | | |  | **J2R approach** |  |  |  | **CIR approach** |  |  |
| **Outcome** | **M diff (95% CI)** | **ES** | **p** |  | **M diff (95% CI)** | **ES** | **p** |  | **M diff (95% CI)** | **ES** | **p** |
| Intuitive eating (IES-3 total) | 0.30 [0.14, 0.46] | 0.54 | <.001 |  | 0.35 [0.20, 0.49] | 0.63 | <.001 |  | 0.31 [0.17, 0.44] | 0.53 | <.001 |
| ED psychopathology (ED15) | -0.45 [-0.73, -0.17] | -0.46 | 0.001 |  | -0.64 [-0.89, -0.38] | -0.66 | <.001 |  | -0.48 [-0.72, -0.25] | -0.56 | <.001 |
| Objective binge eating | -0.10 [-0.39, 0.20] | 0.91 | 0.515 |  | -0.33 [-0.62, -0.03] | 0.72 | 0.028 |  | -0.16 [-0.45, 0.13] | 0.85 | 0.278 |
| Dietary restriction | -0.34 [-0.85, 0.17] | 0.71 | 0.191 |  | -0.72 [-1.18, -0.26] | 0.49 | 0.002 |  | -0.37 [-0.83, 0.09] | 0.69 | 0.117 |
| Compensatory behaviors | -0.25 [-1.01, 0.50] | 0.78 | 0.506 |  | -1.00 [-1.73, -0.27] | 0.37 | 0.007 |  | -0.53 [-1.11, 0.05] | 0.59 | 0.076 |
| Body appreciation (BAS-3SF) | 0.20 [0.23, 0.38] | 0.27 | 0.025 |  | 0.27 [0.09, 0.45] | 0.37 | 0.004 |  | 0.21 [0.03, 0.39] | 0.27 | 0.022 |
| Body interoception (MAIA-2) | 0.29 [0.02, 0.56] | 0.28 | 0.035 |  | 0.30 [0.00, 0.60] | 0.29 | 0.052 |  | 0.29 [0.02, 0.57] | 0.27 | 0.038 |
| LMFC = last mean carried forward; J2R = jump to reference; and CIF = copy increments in reference; mean differences and effect sizes are derive from ITT analysis using multiple imputation. effect size, d, for all outcomes except OBE, SBE compensatory behaviors and restriction (which presents rate ratio). | | | | | | | | | | | |
